# Congenital Absence of the Left Atrial Appendage in Atrial Fibrillation With Gastrointestinal Bleeding

**DOI:** 10.1016/j.jaccas.2026.107764

**Published:** 2026-06-10

**Authors:** Mohamad Amer Nashtar, Martin Steinmetz, Jörn Trippe, Polykarpos Christos Patsalis, Obayda Azizy

**Affiliations:** Department of Cardiology, Angiology and Internal Emergency Medicine, University Hospital Knappschaftskrankenhaus Bochum, Ruhr-University Bochum, Bochum, Germany

**Keywords:** absence of left atrial appendage, anticoagulation therapy, atrial fibrillation, cardiac MRI, gastrointestinal bleeding, Transesophageal echocardiography

## Abstract

**Background:**

Atrial fibrillation (AF) increases the risk of thromboembolic events, most commonly owing to thrombus formation in the left atrial appendage (LAA). Anticoagulation reduces thromboembolic risk but increases bleeding risk. Congenital absence of the LAA is a rare anatomical variant that may influence anticoagulation strategies.

**Case Summary:**

A 78-year-old man with permanent AF and recurrent obscure gastrointestinal bleeding was evaluated for potential LAA occlusion. Transesophageal echocardiography and cardiac magnetic resonance imaging demonstrated congenital absence of the LAA without evidence of atrial thrombus. Considering the patient's high bleeding risk, anticoagulation therapy was discontinued after multidisciplinary assessment. No cardiac thrombus formation was detected on imaging at 6 months, and the patient remained clinically free of thromboembolic events at 18 months.

**Discussion:**

This case illustrates the importance of multimodal imaging in identifying congenital LAA absence and guiding individualized anticoagulation decisions.

**Take-Home Message:**

Congenital LAA absence may allow individualized reconsideration of anticoagulation in selected AF patients with high bleeding risk.

## Visual Summary

**Figure:**
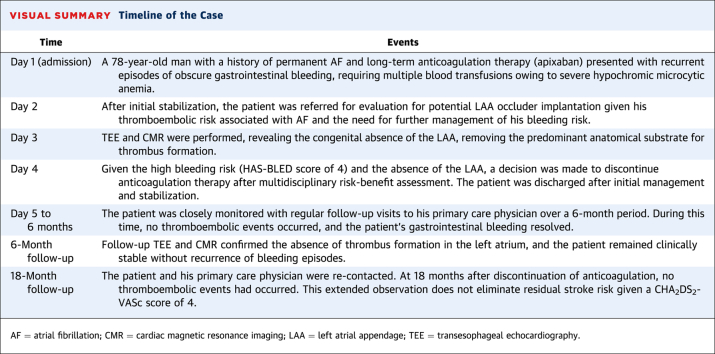

## History of Presentation

A 78-year-old man with a history of permanent atrial fibrillation (AF) and long-term anticoagulation therapy (apixaban) presented with recurrent episodes of obscure gastrointestinal bleeding (GIB), requiring multiple blood transfusions owing to severe hypochromic microcytic anemia. After initial stabilization, the patient was referred for evaluation for potential left atrial appendage (LAA) occluder implantation given the thromboembolic risk associated with AF and the need for further management of his bleeding risk.

**Take-Home Messages**

• The absence of the LAA in AF patients may support individualized reconsideration of anticoagulation in selected patients with high bleeding risk, although residual thromboembolic risk related to atrial and systemic factors persists.
• Multimodal imaging (TEE and CMR) is essential for confirming congenital LAA absence and guiding anticoagulation therapy decisions in such patients.

## Past Medical History

• Permanent AF
• Type 2 diabetes mellitus
• Long-term anticoagulation therapy with apixaban (Eliquis)
• Recurrent obscure GIB
• No history of stroke or thromboembolism

## Differential Diagnosis

• Left atrial thrombus
• Congenital absence of the LAA
• Hypoplastic or atypically positioned LAA

## Investigations

**Figure 1:**
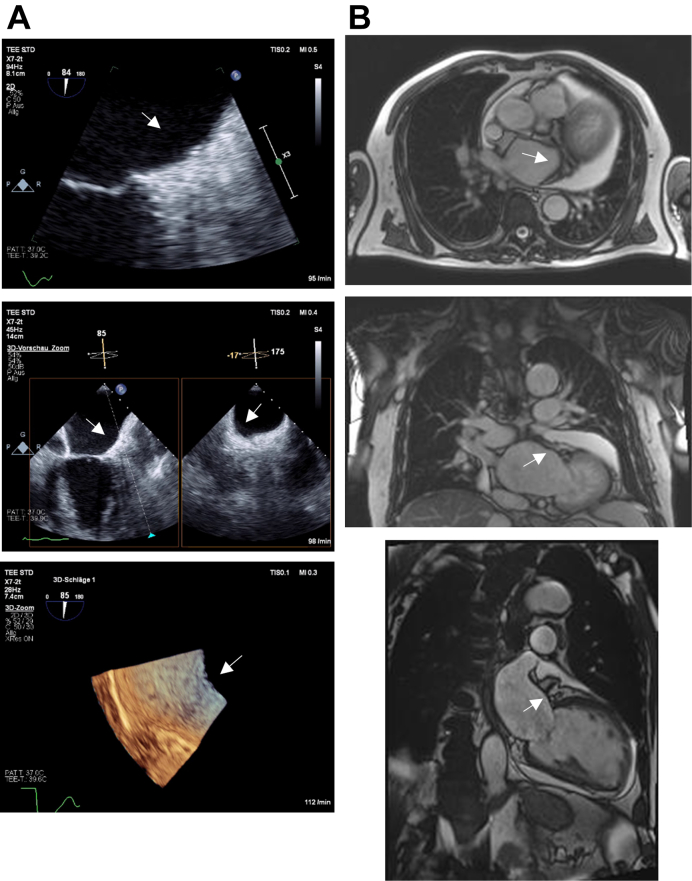
Absence of the Left Atrial Appendage as Demonstrated by Transesophageal Echocardiography and Cardiac Magnetic Resonance Imaging (A) Transesophageal echocardiography images in 3 different views demonstrating the absence of the LAA. (B) Cardiac magnetic resonance imaging confirming the absence of the LAA in 3 distinct views. The arrows mark the region in which the LAA would typically be located. LAA = left atrial appendage.

Transesophageal echocardiography (TEE) revealed the complete absence of the LAA (Figure 1A, Video 1). Additionally, TEE excluded the presence of any thrombus within the left atrium. This finding was further confirmed by cardiac magnetic resonance imaging (CMR), which not only verified the absence of the LAA but also ruled out thrombus formation and neoplastic pathology (Figure 1B, Video 2).

## Management (Medical/Interventional)

Given the patient's high risk of bleeding (HAS-BLED score of 4), a multidisciplinary decision was made to discontinue anticoagulation therapy. This decision was based on the patient's recurrent GIB and the presumed lower risk of thromboembolism given the congenital absence of the LAA, despite a CHA_2_DS_2_-VASc score of 4. Considering the patient's overall risk profile, including both thromboembolic and bleeding risks, the cessation of anticoagulation therapy was deemed appropriate.

After this decision, the patient was closely monitored, with regular follow-up visits to his primary care physician over a 6-month period. During this time, no thromboembolic events occurred. Additionally, the patient's GIB resolved, and he remained clinically stable without any recurrence of bleeding episodes.

## Outcome and Follow-Up

The patient remained clinically stable without embolic complications. Follow-up TEE and CMR at 6 months confirmed the absence of left atrial thrombus. Given the patient's CHA_2_DS_2_-VASc score of 4, this event-free 6-month period must be interpreted cautiously, as the expected annual stroke risk limits the inferential value of short-term follow-up in a single case. We subsequently re-contacted the patient and his primary care physician; at 18 months after discontinuation of anticoagulation, no thromboembolic events have occurred.

## Discussion

The LAA plays a central role in the pathophysiology of thromboembolism in AF, with approximately 91% of left atrial thrombi located in the LAA.^1^ The absence of the LAA is an extremely rare congenital anomaly, with only a few cases documented in the literature.^2^, ^3^, ^4^ In the present case, the congenital absence of the LAA removed the predominant anatomical substrate for thrombus formation in AF. However, thromboembolism in AF is not exclusively confined to the LAA, and thrombus formation may still occur within the atrial body, particularly in patients with systemic vascular risk factors reflected by an elevated CHA_2_DS_2_-VASc score. Therefore, the decision to discontinue anticoagulation was based on an individualized, multidisciplinary risk-benefit assessment in the context of recurrent transfusion-dependent GIB, rather than on the assumption of complete elimination of stroke or systemic thromboembolic risk. While the absence of the LAA significantly reduces thromboembolic risk, this remains a clinically unstandardized scenario, as most guidelines do not address it directly. It has already been shown that LAA morphology can serve as a useful predictor of ischemic stroke risk in patients with AF.^5^ The clinical reasoning in this case may be conceptually compared to percutaneous LAA occlusion, where mechanical exclusion of the appendage from systemic circulation has been shown to allow discontinuation of long-term anticoagulation in selected patients. Randomized trials such as PROTECT AF, PREVAIL, and AMULET IDE are based on the premise that elimination of the LAA as the predominant thrombus source reduces stroke risk to a level comparable to anticoagulation.^6^, ^7^, ^8^ Congenital absence of the LAA may represent a natural anatomical correlate of this principle. However, unlike device-based closure, congenital absence has not been evaluated in prospective outcome studies, and thromboembolic risk cannot be assumed to be equivalent to that observed after successful occlusion. Therefore, clinical decisions must remain individualized.

Diagnostic imaging played a crucial role in this case. Considering the variations in the position and morphology of the LAA, multimodal imaging, including TEE, cardiac computed tomography, and CMR, is often necessary to differentiate true congenital absence from a diminutive or atypically positioned appendage.^9^ TEE is considered the gold standard for evaluating the LAA, providing high-resolution imaging of its structure and flow. CMR offers complementary multiplanar visualization. Given the rarity of congenital LAA absence, no systematic sensitivity data comparing computed tomography, TEE, and CMR for this specific diagnosis are available. In the present case, the absence of a definable LAA ostium or trabeculated cavity across multiple imaging planes on both TEE and CMR supported the diagnosis of true congenital absence and excluded atrial thrombus formation.

This case emphasizes the importance of a tailored, individualized approach to anticoagulation therapy in AF patients, particularly in those at high risk of bleeding. While anticoagulation therapy remains the cornerstone of management for most AF patients, the absence of the LAA represents a unique clinical scenario in which the benefits of anticoagulation may not outweigh the risks of bleeding. In such cases, the decision to discontinue anticoagulation should be guided by a comprehensive risk-benefit assessment, considering both thromboembolic and bleeding risks. Long-term follow-up with imaging techniques, including TEE and CMR, is crucial to ensure the patient's safety and to monitor for any potential thromboembolic events.

Current clinical guidelines, including those from the European Society of Cardiology^10^ and the American Heart Association/American College of Cardiology/Heart Rhythm Society,^11^ recommend oral anticoagulation for patients with AF who have an elevated thromboembolic risk, typically defined as a CHA_2_DS_2_-VASc score of ≥2. However, these guidelines do not specifically address cases of congenital LAA absence, leaving clinicians to rely on individualized decision-making. This case suggests that congenital absence of the LAA may be associated with a lower thromboembolic risk; however, risk is not eliminated. Discontinuation of anticoagulation should therefore be based on individualized risk-benefit assessment, particularly in patients with high bleeding risk. The limited duration of follow-up represents an important limitation. Even with 18 months of event-free observation, conclusions regarding long-term thromboembolic safety cannot be drawn from a single case, particularly in the context of an elevated CHA_2_DS_2_-VASc score. Further studies are needed to better define clinical outcomes and inform anticoagulation strategies in this rare population.

## Conclusions

Congenital absence of the LAA in patients with AF may provide a basis for discontinuing anticoagulation therapy in selected high-risk patients, particularly those with recurrent bleeding complications. Multimodal imaging techniques such as TEE and CMR are critical for confirming this rare anatomical anomaly and guiding anticoagulation management.

## Funding Support and Author Disclosures

The authors have reported that they have no relationships relevant to the contents of this paper to disclose.

## Appendix

Video 1 Transesophageal Echocardiography Demonstrating Congenital Absence of the Left Atrial Appendage Imaging in multiple planes shows absence of a definable left atrial appendage ostium or trabeculated cavity. No left atrial thrombus is visualized.

Video 2 Cardiac Magnetic Resonance Imaging Confirming Congenital Absence of the Left Atrial Appendage Multiplanar cine sequences demonstrate a smooth left atrial contour without evidence of a distinct appendage cavity or neck. No intracardiac thrombus is detected.
